# Integrated Single-Cell and Bulk Transcriptomic Analyses Identify a B Cell- and Plasma Cell-Associated Prognostic Signature and a Candidate Tumor-Suppressive Role for *FUT8* in Ovarian Cancer

**DOI:** 10.3390/genes17070784

**Published:** 2026-07-08

**Authors:** Yiya Wang, Yuan Shi, Ruibin Zhu, Cong Yu, Guoying Wu, Zihan Li, Ju Zhu, Yuxin Lei, Qingqing Wang

**Affiliations:** 1 School of Life Sciences, Qilu Normal University, Jinan 250200, China; shivery@qlnu.edu.cn (Y.S.); ruibin.zhu@qlnu.edu.cn (R.Z.); yc-1127@163.com (C.Y.); guoying.wu@qlnu.edu.cn (G.W.); 13697819469@163.com (J.Z.); a1635067589@126.com (Y.L.); wangqq6266@163.com (Q.W.); 2 College of Life and Geographic Sciences, Kashi University, Kashi 844000, China; 15610948089@163.com

**Keywords:** ovarian cancer, prognostic model, single-cell sequencing, *FUT8*, tumor microenvironment, N-glycosylation

## Abstract

Background: Ovarian cancer (OC) exhibits substantial tumor heterogeneity and an immunosuppressive tumor microenvironment (TME), both contributing to its unfavorable clinical outcomes. Recent studies have increasingly demonstrated that dysregulated glycosylation significantly impacts tumor progression and immune modulation. However, the specific functions and implications of glycosylation-associated regulators in OC remain poorly understood. This study integrates single-cell and bulk transcriptomic data to uncover crucial genes within the TME and investigates the potential role of Fucosyltransferase 8 (*FUT8*) in OC development. Methods: Single-cell RNA sequencing (scRNA-seq) data from OC and normal ovarian tissues (GSE184880, n = 12) were analyzed using Seurat and Harmony for clustering and annotation. Ro/e analysis identified B cells and plasma cells as enriched immune populations. Their marker genes were integrated with The Cancer Genome Atlas (TCGA) cohort as the training set, while internal testing and an independent external validation cohort (GSE63885) were used to construct and validate the prognostic model. *FUT8* function was evaluated in OC cell lines using quantitative real-time PCR (qRT-PCR), cell counting kit-8 (CCK-8) assays, flow cytometry, and transcriptomic sequencing. Gene Set Variation Analysis (GSVA), Gene Set Enrichment Analysis (GSEA), and virtual knockout analyses were performed to explore *FUT8*-associated pathways. Results: We constructed a single-cell atlas consisting of 46,235 cells classified into seven principal cell populations, highlighting significant enrichment of B and plasma cells in OC tissues. The prognostic signature could stratify patients into high- and low-risk groups across training, internal validation, and external validation cohorts, showing consistent prognostic stratification capacity. *FUT8* expression was elevated in OC samples and was associated with favorable overall survival (OS). Experimental overexpression of *FUT8* in OC cell lines resulted in reduced cell proliferation and increased apoptosis. Both transcriptomic analyses and virtual knockout studies consistently associated *FUT8* with pathways related to N-glycosylation. High-risk patients exhibited predicted activation of Wnt/β-catenin, Hedgehog, and Kras pathways, coupled with diminished immune cell infiltration. Conclusions: We developed a prognostic signature informed by single-cell data for OC and identified *FUT8* as a potential regulator associated with N-glycosylation processes in OC. These results offer insights into OC molecular characteristics and highlight *FUT8* as a candidate biomarker with potential prognostic relevance. Further experimental studies are necessary to validate and elucidate the precise molecular mechanisms involved.

## 1. Introduction

OC remains one of the most lethal gynecological cancers and significantly threatens global women’s health [1]. The majority of patients present with advanced-stage disease (International Federation of Gynecology and Obstetrics (FIGO) III/IV), resulting in a poor prognosis and low 5-year OS rates [2]. OC is highly heterogeneous and includes multiple histological subtypes such as high-grade serous ovarian carcinoma (HGSOC), endometrioid carcinoma, clear cell carcinoma, and mucinous carcinoma, each varying in molecular features and clinical outcomes [3]. HGSOC is particularly aggressive, accounting for the majority of OC-related mortality due to its frequent recurrence and development of resistance to platinum-based chemotherapy [4]. Cytoreductive surgery combined with platinum-based chemotherapy remains the standard first-line treatment, but recurrence is common, and treatment options for relapsed disease are limited [5]. Platinum resistance contributes significantly to therapeutic failure and poor survival outcomes [6,7]. Clinical management challenges in OC include the absence of reliable early detection biomarkers [8], modest efficacy of immune checkpoint inhibitors [7], and limited prognostic accuracy of traditional tools such as FIGO staging and serum cancer antigen 125 (CA125) [9]. Despite potential prognostic or diagnostic relevance, biomarkers like human epididymis protein 4 (*HE4*), growth differentiation factor 15 (*GDF15*), and Homeobox A1 (*HOXA1*) are constrained by OC heterogeneity [10,11,12]. Therefore, identifying new prognostic biomarkers and therapeutic targets is critical for advancing personalized patient care.

The TME is heterogeneous and critically linked to therapeutic resistance and patient prognosis in OC. The OC TME includes diverse immune and stromal cell populations, such as cytotoxic T lymphocytes (CTLs), regulatory T cells (Tregs), and other cellular subsets, which collectively influence tumor progression, immune surveillance, and evasion [13]. Bulk transcriptome sequencing methods typically reflect average gene expression across cell types, obscuring intratumoral heterogeneity at the single-cell level [14]. scRNA-seq overcomes this limitation, facilitating detailed mapping of TME cellular landscapes, identification of rare cell populations, and discovery of critical regulatory genes [15]. Recent scRNA-seq studies have provided significant insights into OC cellular heterogeneity, immune regulation, and disease progression [16,17,18,19]. Integrating single-cell with spatial transcriptomics has further emphasized the importance of epithelial–stromal interactions, immune heterogeneity, and immune escape mechanisms [12].

Among immune cells, cytotoxic T cells and macrophages are well-studied, yet emerging evidence underscores that B cells and plasma cells also play important roles in anti-tumor immunity through antibody production, antigen presentation, and formation of tertiary lymphoid structures (TLS) [20,21]. The presence of these humoral immune components is associated with improved prognostic outcomes and enhanced antitumor responses across various cancers [22,23]. Emerging evidence further indicates that B/plasma cell functional states, supported by follicular helper T cells (Tfh) during TLS maturation, may provide prognostic information beyond immune infiltration levels alone. This suggests their potential relevance for tumor stratification in OC, particularly HGSOC [24,25,26].

In this study, we integrated scRNA-seq data with bulk transcriptomic data. Ro/e analysis indicated that B and plasma cells were enriched immune subsets within the OC TME. Using LASSO–Cox regression based on cell-type-specific marker genes, we created a prognostic model. *FUT8* emerged as a gene of particular interest within the prognostic signature due to its protective role, substantial contribution to risk stratification, and correlation with patient survival. *FUT8* encodes α1,6-fucosyltransferase, the sole enzyme mediating core fucosylation, a critical modification in protein N-glycosylation [27]. *FUT8* showed heterogeneous expression across multiple TME cell populations, suggesting that it may function as a tumor-intrinsic glycosylation-related regulator broadly expressed across TME cell types. Although *FUT8* is not a B-cell-specific lineage marker, it was originally identified from the B/plasma cell-derived prognostic signature. Accumulating evidence indicates that core fucosylation modulates N-glycosylation of immune cell surface receptors, including the B-cell receptor, and thereby regulates immune receptor signaling pathways. These findings suggest a potential functional link between glycosylation and immune regulation [28]. Meanwhile, *FUT8* expression in tumor epithelial cells also indicates a possible tumor-intrinsic role in glycosylation regulation, which may shape the immune microenvironment. Because the role of *FUT8* in OC remains largely unclear and context-dependent, we selected *FUT8* as a key candidate gene linking the prognostic signature, glycosylation regulatory mechanisms, and functional validation in OC cells. Therefore, we further investigated the biological role of *FUT8* using functional experiments and transcriptomic analyses. We also explored the relationship between the prognostic model, immune infiltration, and pathway activity and constructed a nomogram for survival prediction. Our findings provide new insights into the relationship among B cell-associated molecular features, glycosylation-related processes, and the TME.

## 2. Materials and Methods

### 2.1. Data Acquisition and Quality Control

scRNA-seq data from 12 OC specimens, consisting of seven tumor and five normal ovarian tissues, were acquired from the publicly accessible Gene Expression Omnibus (GEO) database (National Center for Biotechnology Information, Bethesda, MD, USA; https://www.ncbi.nlm.nih.gov/geo; accessed on 14 February 2025) under accession GSE184880 [29]. This dataset encompassed seven treatment-naive HGSOC samples across different disease stages and five non-malignant ovarian controls matched by age. Preprocessing and quality control steps were performed using the Seurat R package (version 4.3.0; Satija Lab, New York Genome Center, New York, NY, USA) [30]. Proportions of mitochondrial and ribosomal RNA genes were quantified per cell. Cells satisfying the following criteria were retained: nFeature_RNA ≥ 500, nFeature_RNA, nCount_RNA, and mitochondrial gene content (percent.mt) within median ± 3 × median absolute deviation (MAD). DoubletFinder (version 2.0.4; McGinnis et al., University of California, San Francisco, CA, USA) was utilized to identify and exclude doublets. After these stringent filtering processes, 46,235 high-quality cells remained for downstream analysis (Table S1). Additional bulk transcriptomic and survival data were downloaded from the GSE63885 dataset (GPL570 platform), where 75 patients with complete survival records were included following integration of expression data with clinical metadata [31]. Furthermore, preprocessed expression data for 429 OC samples were obtained from the TCGA database (National Cancer Institute, Bethesda, MD, USA; https://cancergenome.nih.gov; accessed on 14 February 2025) [32].

### 2.2. Data Standardization and Cell Annotation

The Seurat R package (version 4.3.0) LogNormalize method was applied to normalize gene expression counts. Cell cycle phases were assessed using CellCycleScoring, and highly variable genes were identified through the FindVariableFeatures function. To mitigate potential confounding effects, mitochondrial gene percentages and cell cycle scores were regressed out during data scaling. Harmony (version 1.2.4; Korsunsky et al., Harvard Medical School, Boston, MA, USA) was implemented to correct batch effects, followed by linear dimensionality reduction using Principal Component Analysis (PCA), with the first 20 principal components selected for further analysis. Uniform Manifold Approximation and Projection (UMAP) facilitated nonlinear dimensionality reduction, and clustering was conducted with a resolution setting of 0.2. Cell identities were primarily annotated based on previous literature and the CellMarker database (version 2.0; http://117.50.127.228/CellMarker/; accessed on 9 March 2025), with automated SingleR package (version 2.0.0; Bioconductor, Seattle, WA, USA) annotation providing additional validation [33,34].

### 2.3. Ro/e Enrichment Analysis

Relative cell type enrichment in tumor tissues compared to normal controls was quantified by calculating the observed-to-expected (Ro/e) cell ratio. A Ro/e > 1 indicated cellular enrichment, whereas a Ro/e < 1 implied depletion.

### 2.4. Model Construction and Prognosis

To identify prognosis-associated genes, univariate Cox regression analyses were first performed (*p* < 0.05). Prognostic modeling then utilized LASSO–Cox regression via the glmnet R package (version 4.1-7; Friedman laboratory, Stanford University, Stanford, CA, USA). Patient-specific risk scores were computed using the following formula: risk score = Σ (gene expression level × LASSO coefficient). Based on median risk scores, patients were stratified into high-risk and low-risk groups. To reduce the potential instability caused by a single random split, repeated random 4:1 resampling (10,000 iterations) was performed during the exploratory modeling stage. In each iteration, univariate Cox regression and LASSO–Cox regression were reapplied in the training set, and model performance was then evaluated in the corresponding internal testing set. The final 13-gene signature was derived from a single prespecified 4:1 training/testing split with a fixed random seed that showed consistent prognostic discrimination in the training set and internal testing set, rather than being determined solely by the highest area under the curve (AUC) from repeated iterations. Kaplan–Meier survival curves were generated to compare OS between groups, with statistical significance assessed by log-rank tests. Multivariate Cox regression analyses verified the independent prognostic value of the risk score beyond clinical variables. Time-dependent receiver operating characteristic (ROC) curves were generated, and area under the curve (AUC) metrics at 2, 3, and 5 years were evaluated to assess model accuracy.

### 2.5. External Validation of the Prognostic Model

The GSE63885 dataset, derived from the GPL570 microarray platform, provided preprocessed expression data directly utilized in this study. Probe identifiers were converted to official gene symbols based on the platform’s annotation files. In instances where multiple probes corresponded to a single gene symbol, their mean expression was calculated for each sample. After mapping gene identifiers, all 13 genes comprising the prognostic signature were verified as detectable within the GSE63885 dataset. Risk scores for external validation were computed strictly using the identical formula and LASSO-derived coefficients obtained from the TCGA training cohort, without model re-fitting. Due to inherent platform-specific expression differences between TCGA RNA-seq and GSE63885 microarray data, patients within the validation cohort were categorized into high- and low-risk groups using cohort-specific median risk scores. Prognostic model performance in the validation cohort was evaluated consistently through previously described survival analysis methods and time-dependent AUC metrics.

### 2.6. Functional Validation of the Key Prognostic Gene FUT8

The biological significance of *FUT8* in OC was explored via integrated bioinformatics analysis, tissue validation, and in vitro experimental approaches. *FUT8* expression levels and associated prognostic value were assessed using TCGA, GTEx, and Kaplan–Meier analyses. Immunohistochemistry (IHC) evaluated FUT8 protein expression in OC tissues versus adjacent normal tissues, while qRT-PCR and Western blotting (WB) quantified *FUT8* mRNA and protein in immortalized ovarian surface epithelial cells (IOSE-80) and OC cell lines. *FUT8* was overexpressed in cells using pcDNA3.1-FUT8 plasmid transfection, and subsequent cellular proliferation, cell cycle dynamics, and apoptosis were examined via CCK-8 assay, PI/RNase staining, and Annexin V-FITC/PI staining assays, respectively. RNA sequencing of *FUT8*-overexpressed A2780 cells, alongside bioinformatics analyses and qRT-PCR validation, identified pathways related to *FUT8* function. Additionally, virtual knockout analysis was conducted to investigate *FUT8*-associated biological mechanisms within single-cell regulatory networks. All in vitro cellular experiments were performed in three independent biological replicates to ensure reproducibility. Specific experimental methods are detailed in the Supplementary Materials.

### 2.7. Immune Cell Infiltration and Drug Sensitivity Analysis

The single-sample gene set enrichment analysis (ssGSEA) method quantified 29 immune cell subpopulations to evaluate immune cell infiltration in OC tissues [35]. Immune infiltration analyses were performed using the GSVA R package (version 1.46.0; Bioconductor, Castelo et al., Barcelona, Spain). ESTIMATE was employed to compute tumor purity, stromal, immune, and ESTIMATE scores. The Wilcoxon rank-sum test was used to analyze differences in immune cell profiles between risk groups. Multiple testing correction across the 29 immune cell subpopulations was performed, and adjusted *p*-values < 0.05 were considered statistically significant.

The oncoPredict R package (version 0.2; Maes et al., University of Minnesota, Minneapolis, MN, USA), integrating data from the Genomics of Drug Sensitivity in Cancer (GDSC) database (GDSC2, release 8.5; Wellcome Sanger Institute, Cambridge, UK; https://www.cancerrxgene.org/; accessed on 15 March 2025), predicted chemotherapy sensitivity in individual tumors. Elastic net regression with tenfold cross-validation on the GDSC dataset estimated half-maximal inhibitory concentration (IC_50_) values for various chemotherapeutic drugs. Batch effects were adjusted using the sva R package (version 3.46.0; Bioconductor, Leek Lab, Johns Hopkins University, Baltimore, MD, USA) using the ComBat algorithm, and the Wilcoxon rank-sum test was applied to evaluate differences in IC_50_ values between risk groups. After multiple testing corrections, adjusted *p*-values < 0.05 were considered statistically significant.

### 2.8. Nomogram Model Construction

An integrated nomogram was developed using independent prognostic variables identified by multivariate Cox regression, including the derived risk score. Utilizing the rms package (version 6.6-0; Harrell Lab, Vanderbilt University, Nashville, TN, USA) [36], this graphical tool assigns weighted scores to each prognostic factor, cumulatively estimating survival probabilities. Calibration plots assessed the accuracy between predicted and observed clinical outcomes.

### 2.9. GSVA and GSEA

GSVA was conducted using the GSVA R package with annotated gene sets from the Molecular Signatures Database (MSigDB, v7.0; Broad Institute, Cambridge, MA, USA; https://www.gsea-msigdb.org/gsea/msigdb/; accessed on 15 March 2025) to identify pathway-level changes [35]. Differentially enriched pathways between high- and low-risk groups were recognized. Multiple testing correction was applied, and pathways with adjusted *p*-values < 0.05 were considered significantly altered. Similarly, GSEA utilized MSigDB gene sets to discern biological processes significantly enriched between the defined risk groups [37]. Significant enrichment was considered for pathways when the corrected *p*-value was less than 0.05.

### 2.10. Pseudotime Trajectory Analysis

The Monocle R package (version 2.30.0; Bioconductor, Trapnell Lab, University of Washington, Seattle, WA, USA) was employed to perform pseudotime trajectory analysis, reconstructing hypothetical differentiation pathways. Dynamic expression patterns of prognostic signature genes along these trajectories were analyzed to reveal differentiation-associated variations.

### 2.11. Transcription Factor Regulatory Network Analysis with SCENIC

Single-cell regulatory network inference and clustering (SCENIC) was implemented using the SCENIC R package (version 1.18.0; Aibar et al., VIB Center for Brain & Disease Research, Leuven, Belgium) to reconstruct transcription factor (TF) regulatory networks assessing regulon activity at the single-cell level [38]. The GENIE3 algorithm implemented in the GENIE3 R package (version 1.24.0; Huynh-Thu et al., University of Liège, Liège, Belgium) was used to identify TF-target co-expression modules, and significant regulons were selected through motif enrichment analysis. Regulon activities were subsequently quantified per cell to characterize distinct cellular states and regulatory dynamics across subpopulations.

### 2.12. Statistical Analysis

All statistical analyses were performed using R software (version 4.3.0; R Foundation for Statistical Computing, Vienna, Austria) and IBM SPSS Statistics (version 26.0; IBM Corp., Armonk, NY, USA). Differential expression analysis in scRNA-seq data was performed using the Seurat R package (version 4.3.0; FindMarkers/FindAllMarkers functions), based on the Wilcoxon rank-sum test by default. Gene expression differences between two groups were evaluated using the Wilcoxon rank-sum test, and comparisons among multiple groups were performed using the Kruskal–Wallis test. Functional enrichment analyses (Gene Ontology (GO) and Kyoto Encyclopedia of Genes and Genomes (KEGG)) were performed using the hypergeometric test or Fisher’s exact test. Survival differences between groups were assessed using the log-rank test. Hazard ratios (HRs) and 95% confidence intervals (CIs) were calculated using Cox proportional hazards regression models. Correlation analyses were performed using Spearman correlation. All in vitro experiments were independently repeated at least three times. Data are presented as mean ± standard error of the mean (SEM). Statistical significance was defined as *p* < 0.05 (* *p* < 0.05, ** *p* < 0.01, *** *p* < 0.001).

## 3. Results

### 3.1. Single-Cell Transcriptomic Landscape and Cell Type Annotation

**Figure 1:**
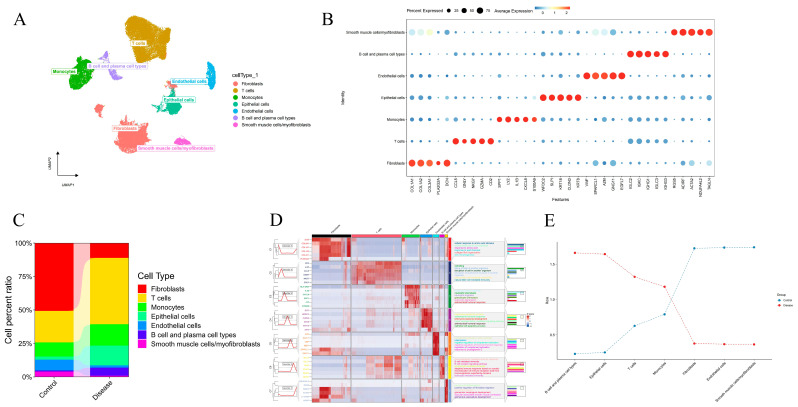
Cell type annotation and identification of core cellular components in the OC TME. (**A**) UMAP visualization delineating 12 cell clusters grouped into seven principal cell categories. (**B**) Bubble plot illustrating the expression of canonical markers specific to each cell type. (**C**) Comparative analysis of cell-type composition between OC and normal ovarian tissues. (**D**) Differential pathway enrichment analysis across the seven cell types, with statistical significance assessed using the Wilcoxon rank-sum test (adjusted *p* < 0.05). (**E**) Ro/e analysis indicating cell-type enrichment (Ro/e > 1) or depletion (Ro/e < 1) in OC tissues. A total of 46,235 single cells from 12 samples (7 tumor and 5 normal ovarian tissues) were analyzed.

Following rigorous quality control, 46,235 single cells from 7 OC samples and 5 normal tissues remained for analysis (Figure S1A–C). Data normalization, dimensionality reduction, and batch-effect correction were implemented via the Harmony algorithm (Figure S1D–G). Cells were categorized into seven primary clusters: T cells, fibroblasts, epithelial cells, monocytes, B/plasma cells, endothelial cells, and smooth muscle/myofibroblast cells (Figure 1A), with canonical markers illustrated in Figure 1B.

### 3.2. Differential Enrichment and TME Composition

To characterize TME remodeling in OC, we compared the relative abundance of major cell populations between normal ovarian and OC tissues. Fibroblasts were the predominant population in normal tissues, followed by T cells and monocytes. In contrast, OC tissues showed a marked shift in cellular composition, with T cells becoming the dominant population and epithelial cells, B cells, and plasma cells also increasing (Figure 1C). Functional enrichment analysis revealed cell-type-specific programs, including collagen fibril organization in fibroblasts, NK cell-mediated cytotoxicity in T cells, neutrophil and granulocyte chemotaxis in monocytes, apoptotic processes in epithelial cells, complement activation in endothelial cells, B cell receptor signaling in B cells/plasma cells, and positive regulation of fibroblast migration in smooth muscle cells/myofibroblasts (Figure 1D). Ro/e analysis further confirmed the preferential enrichment of B cells and plasma cells in OC tissues, indicating an increased humoral immune component within the OC TME (Figure 1E).

### 3.3. Development and Assessment of the Prognostic Model

**Figure 2:**
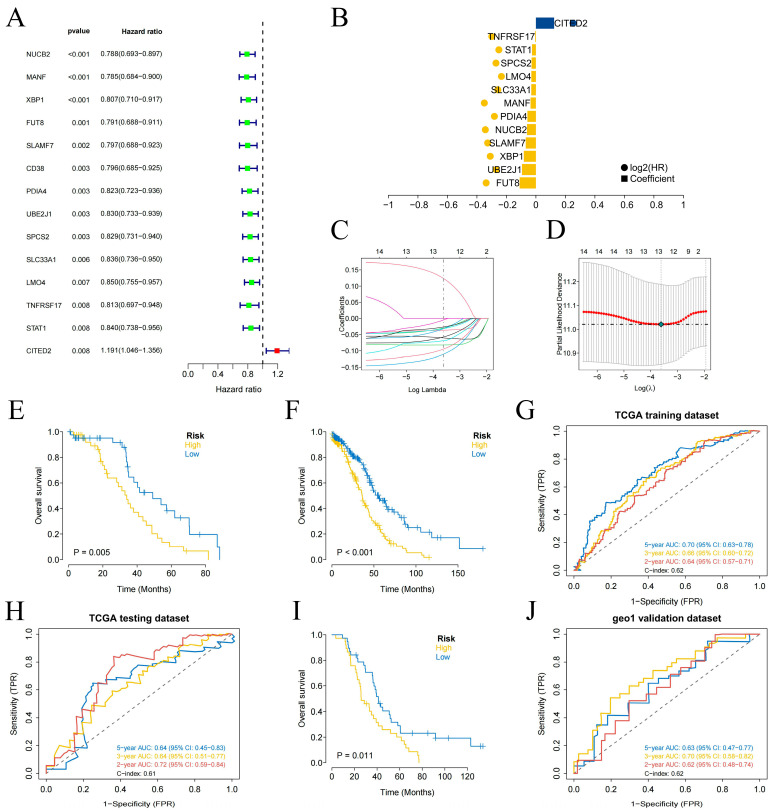
Development and evaluation of the prognostic risk model based on B cell- and plasma cell-specific marker genes. (**A**) Identification of candidate prognostic genes via univariate Cox regression from B and plasma cell-specific marker genes. Hazard ratios and *p* values were estimated using Cox regression analysis. (**B**) LASSO coefficient profiles for selected prognostic genes. (**C**) Optimal λ selection using tenfold cross-validation in the LASSO model. (**D**) Visualization of gene coefficients derived from the LASSO regression. (**E**,**F**) Kaplan–Meier survival curves comparing OS between high- and low-risk groups in the TCGA training and testing cohorts. Survival differences were assessed using the log-rank test. (**G**,**H**) Time-dependent ROC analyses at 2, 3, and 5 years for TCGA cohorts. (**I**) Kaplan–Meier survival curve validation in the independent GEO validation cohort. (**J**) Time-dependent ROC curves for the GEO validation cohort at 2, 3, and 5 years. Cox proportional hazards models were used for survival analyses, and AUC values were calculated using time-dependent ROC analysis. A total of 429 TCGA samples and 75 GEO samples were included in survival analyses.

To construct a prognostic signature based on B cell- and plasma cell-specific marker genes within the TME, specific marker genes were first identified from single-cell transcriptomic data. Subsequently, 177 validated marker genes served as candidate inputs for model construction. Using the TCGA dataset, univariate Cox regression identified 14 of these genes as significantly prognostic in OC patients (*p* < 0.01) (Figure 2A). To assess model stability, repeated random 4:1 resampling (10,000 iterations) was performed as an exploratory step. In each iteration, the TCGA cohort was randomly divided into training and internal testing sets, and univariate Cox regression followed by LASSO–Cox regression was conducted in the training set. This procedure was used to evaluate the robustness of candidate gene selection and model performance across different data splits. The final 13-gene prognostic signature was derived from a single prespecified 4:1 training/testing split using a fixed random seed, which demonstrated consistent prognostic performance in both the training and internal testing cohorts. The final model was not selected based on the highest AUC across resampling iterations. The representative model presented in the main text was constructed using LASSO–Cox regression and included 13 genes (Figure 2B–D). Risk scores were calculated as follows:

Risk Score = *FUT8* × (−0.1105) + *UBE2J1* × (−0.0945) + *XBP1* × (−0.0818) + *SLAMF7* × (−0.0687) + *NUCB2* × (−0.0603) + *PDIA4* × (−0.0575) + *MANF* × (−0.0363) + *SLC33A1* × (−0.0316) + *LMO4* × (−0.0312) + *SPCS2* × (−0.0294) + *STAT1* × (−0.0263) + *TNFRSF17* × (−0.0050) + *CITED2* × (0.1235).

Patients were grouped into high- and low-risk categories based on median risk scores. Kaplan–Meier analyses indicated that high-risk patients exhibited worse OS outcomes in both training and internal testing cohorts (Figure 2E,F). The prognostic accuracy of the signature was further assessed through time-dependent ROC curves, indicating moderate discriminative capacity (Figure 2G,H). External validation in the GSE63885 cohort further supported the consistency of the model’s prognostic trend, with performance metrics comparable to those obtained in internal validation (Figure 2I,J).

### 3.4. Single-Cell Pseudotime and Transcriptional Regulatory Network Analyses

Pseudotime analysis revealed a continuous differentiation trajectory from B cells toward plasma cells, indicating progressive B/plasma cell state transitions within the TME (Figure S2). Along this trajectory, the 13 signature genes exhibited dynamic expression changes associated with B/plasma cell state transitions. SCENIC analysis was further performed to characterize transcriptional regulatory programs during this process. Compared with B cells, plasma cells showed increased activity of multiple transcription factor regulons, including *ATF6*, *CREB3L2*, *ATF4*, *FOXO3*, and *ERF*, which is consistent with enhanced endoplasmic reticulum stress and unfolded protein response activity during plasma cell differentiation (Figure S3). AUCell analysis further indicated functional differences between cell states, with plasma cells showing enrichment of protein processing and stress-response–related pathways (Figure S4). These findings suggest that the B/plasma cell-associated prognostic signature may reflect not only changes in immune cell abundance but also plasma cell differentiation, protein-folding activity, and stress-adaptive regulatory programs.

### 3.5. Cellular Expression Landscape of Signature Genes and Selection of the Core Gene FUT8

**Figure 3:**
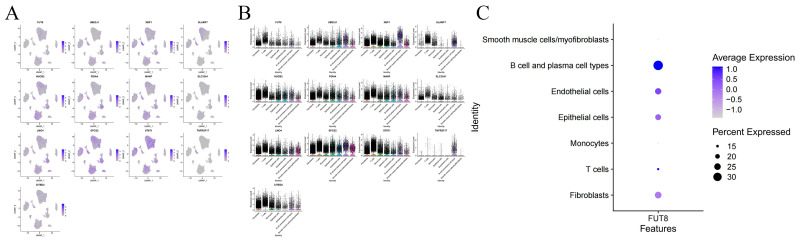
Single-cell expression landscape of the prognostic signature genes. (**A**) UMAP feature plots demonstrating single-cell expression patterns of 13 prognostic genes. (**B**) Violin plots displaying comparative expression levels of the 13 model genes among seven principal OC TME cell types. Differences in gene expression were assessed using the Wilcoxon rank-sum test. (**C**) Dot plot summarizing the expression frequency and intensity of *FUT8* across cell clusters; dot size indicates expression frequency, while the color scale denotes average gene expression. A total of 46,235 single cells from 12 samples (7 tumor and 5 normal ovarian tissues) were analyzed.

To select a gene for detailed validation, we examined the expression patterns of all signature genes across identified cell types. *FUT8* emerged as a candidate due to its notable expression in immune populations, its contribution within the prognostic model, and its known role as the sole enzyme facilitating core fucosylation in N-glycosylation processes. Single-cell analysis revealed heterogeneous *FUT8* expression across immune, stromal, and epithelial compartments (Figure 3A,B). Specifically, B cells and plasma cells exhibited the highest average *FUT8* expression (Figure 3C).

### 3.6. Identification and Expression Validation of FUT8 in OC

**Figure 4:**
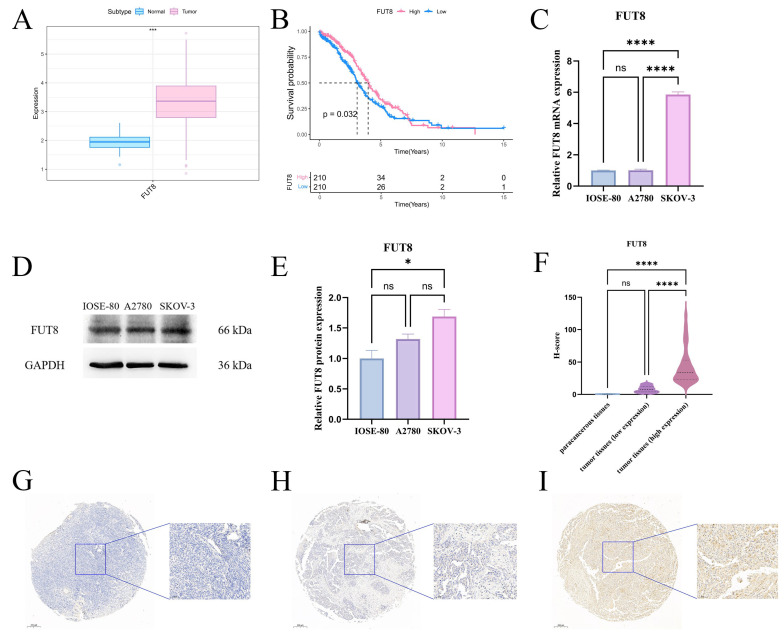
*FUT8* expression and prognostic significance in OC. (**A**) Comparative analysis of *FUT8* mRNA expression between OC and normal tissues. Statistical significance was assessed using the Wilcoxon rank-sum test. (**B**) Kaplan–Meier plot assessing OS based on *FUT8* expression levels in OC patients. Survival differences were evaluated using the log-rank test. (**C**) mRNA expression of *FUT8* in IOSE-80 versus OC cell lines (A2780, SKOV-3) (n = 3). Statistical significance was assessed using one-way ANOVA. (**D**) WB analysis of FUT8 protein levels in IOSE-80, A2780, and SKOV-3 cells (n = 3). (**E**) Quantitative protein expression analysis of FUT8 (n = 3). Statistical significance was assessed using one-way ANOVA. (**F**) H-score quantification comparing FUT8 immunohistochemical staining intensity between adjacent normal and OC tissues. (**G**–**I**) Representative immunohistochemical staining images for FUT8 (×200 magnification). Results are presented as mean ± SEM. “ns” indicates *p* > 0.05, “*” indicates *p* < 0.05, “***” indicates *p* < 0.001, and “****” indicates *p* < 0.0001.

Comparative analysis of OC and normal ovarian tissues from combined TCGA and GTEx datasets confirmed significantly elevated *FUT8* expression in OC (Figure 4A). Lower *FUT8* expression correlated with poorer OS (*p* = 0.032) (Figure 4B), although the separation of survival curves was modest, suggesting limited prognostic power when *FUT8* is considered individually. At the cellular level, IOSE-80 displayed significantly lower *FUT8* mRNA and protein levels compared to OC cell lines (A2780, SKOV-3; Figure 4C–E). Immunohistochemical analysis further validated stronger cytoplasmic FUT8 staining in OC tissues relative to adjacent normal ovarian tissues (Figure 4F–I).

### 3.7. FUT8 Overexpression Suppresses Malignant Phenotypes in OC Cells

**Figure 5:**
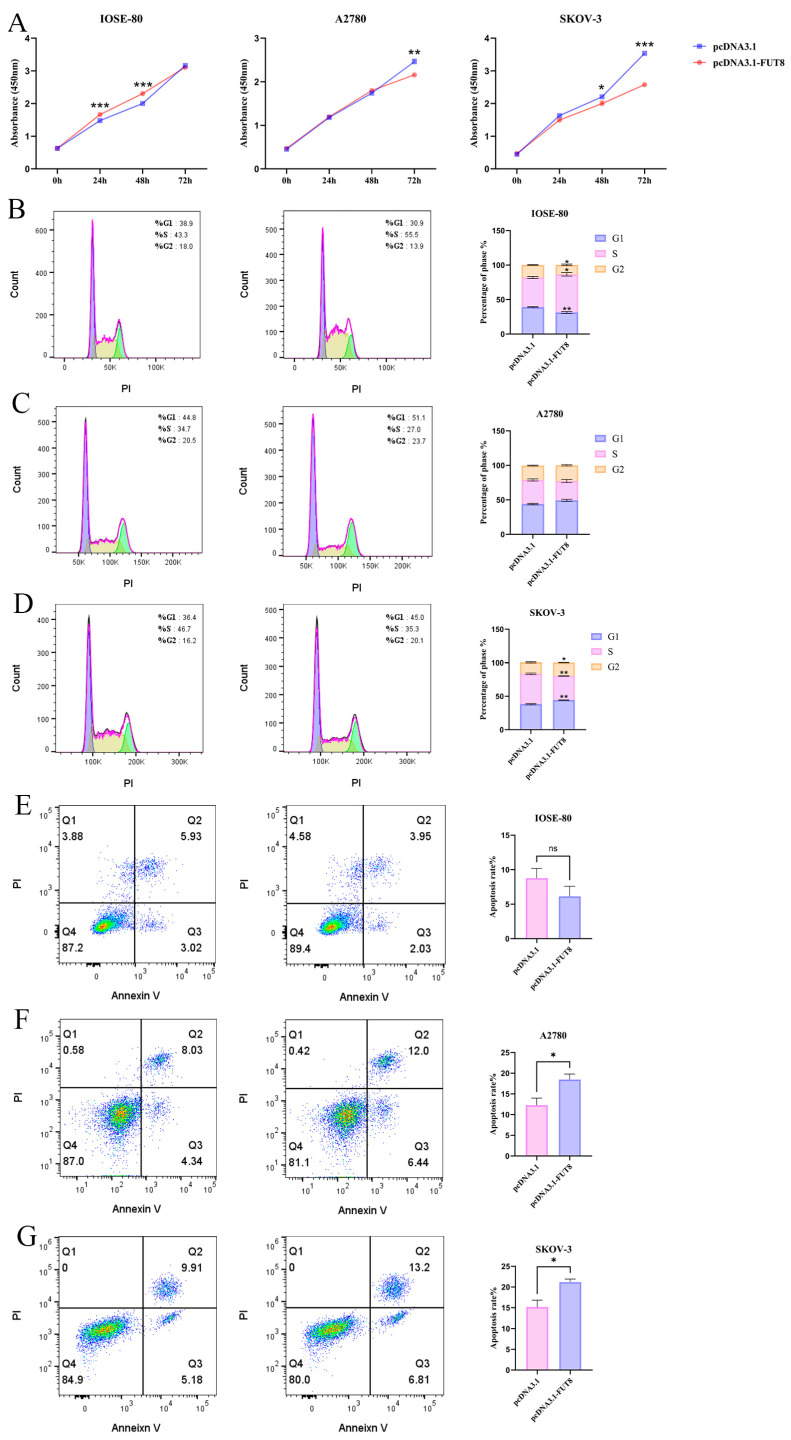
*FUT8* overexpression suppresses proliferation and induces apoptosis in OC cells. (**A**) CCK-8 proliferation assays in IOSE-80, A2780, and SKOV-3 cells with or without *FUT8* overexpression. (**B**–**D**) Flow cytometric analyses of cell cycle phases. (**E**–**G**) Apoptosis assessment via Annexin V/PI staining (n = 3). Statistical significance was assessed using *t*-test. Results are presented as mean ± SEM. “ns” indicates *p* > 0.05, “*” indicates *p* < 0.05, “**” indicates *p* < 0.01, and “***” indicates *p* < 0.001.

*FUT8* overexpression suppressed proliferation in A2780 and SKOV-3 cells, as shown by the CCK-8 assay, but not in IOSE-80 cells (Figure 5A). Flow cytometry demonstrated increased S-phase in IOSE-80 cells but reduced S-phase populations in OC cells (Figure 5B–D). Apoptosis rates were significantly elevated in OC cell lines overexpressing *FUT8*, while IOSE-80 cells showed minimal changes (Figure 5E–G). qRT-PCR analyses confirmed *FUT8*-mediated modulation of proliferation (CCNA1, PCNA) and apoptosis markers (Bcl-2, Bax) (Figure S5).

### 3.8. Transcriptomic Analysis Reveals Potential FUT8-Associated Glycosylation and Immune Regulatory Signatures

**Figure 6:**
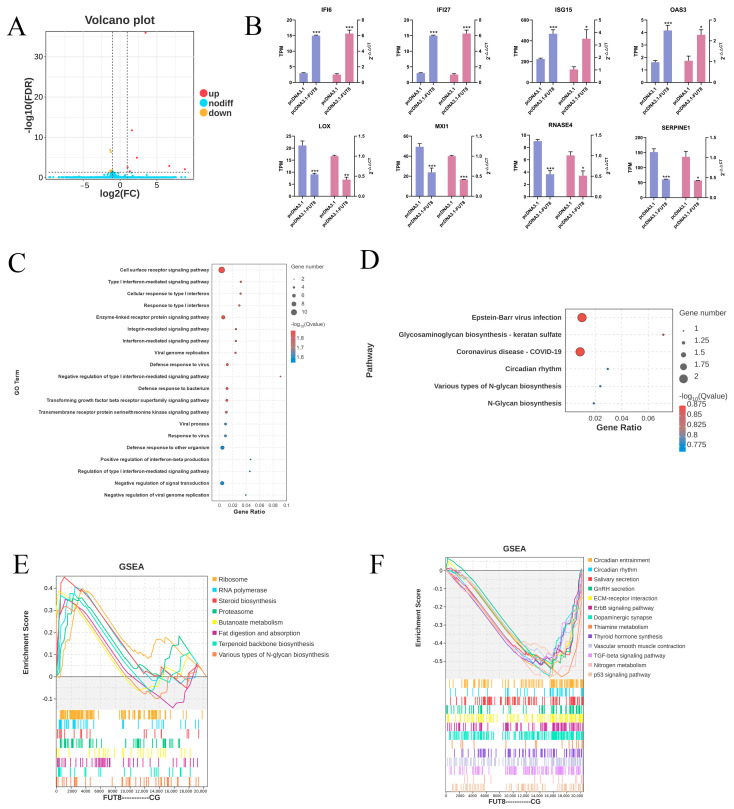
Transcriptomic analysis of *FUT8* overexpression and in silico knockout. (**A**) Volcano plot identifying DEGs in A2780 cells with *FUT8* overexpression (n = 3). DEGs were identified using standard transcriptomic differential expression analysis with thresholds of FDR < 0.05 and |log_2_FC| > 1. (**B**) qRT-PCR validation of representative DEGs (n = 3). Statistical significance was assessed using *t*-test. Results are presented as mean ± SEM. “*” indicates *p* < 0.05, “**” indicates *p* < 0.01, and “***” indicates *p* < 0.001. (**C**,**D**) GO (**C**) and KEGG (**D**) enrichment analyses of DEGs. (**E**,**F**) GSEA in FUT8-overexpressing A2780 cells.

RNA sequencing of *FUT8*-overexpressing A2780 cells identified 17 differentially expressed genes (DEGs) (Table S2), of which 8 were validated by qRT-PCR (Figure 6A,B). Given the limited number of DEGs, DEG-based functional enrichment analysis was interpreted as exploratory. This analysis identified immune-related GO terms, including type I interferon signaling, and glycosylation-associated KEGG pathways, including N-glycan biosynthesis and glycosaminoglycan biosynthesis–keratan sulfate (Figure 6C,D; Table S3). To complement the DEG-centered analysis and reduce bias from the small DEG set, GSEA was performed using the full transcriptomic profile. This analysis suggested enrichment of extracellular matrix (ECM)–receptor interaction and N-glycan biosynthesis pathways (Figure 6E,F; Table S4). In silico virtual knockout analyses provided additional support for these potential associations, showing perturbation of hexose catabolic processes and protein N-linked glycosylation (Figure S6). Collectively, these convergent analyses suggest that *FUT8*-associated glycosylation remodeling may be associated with the regulation of malignant phenotypes in OC cells. Further validation at the protein and functional levels is warranted.

### 3.9. Immune Infiltration and Immunotherapy Response

**Figure 7:**
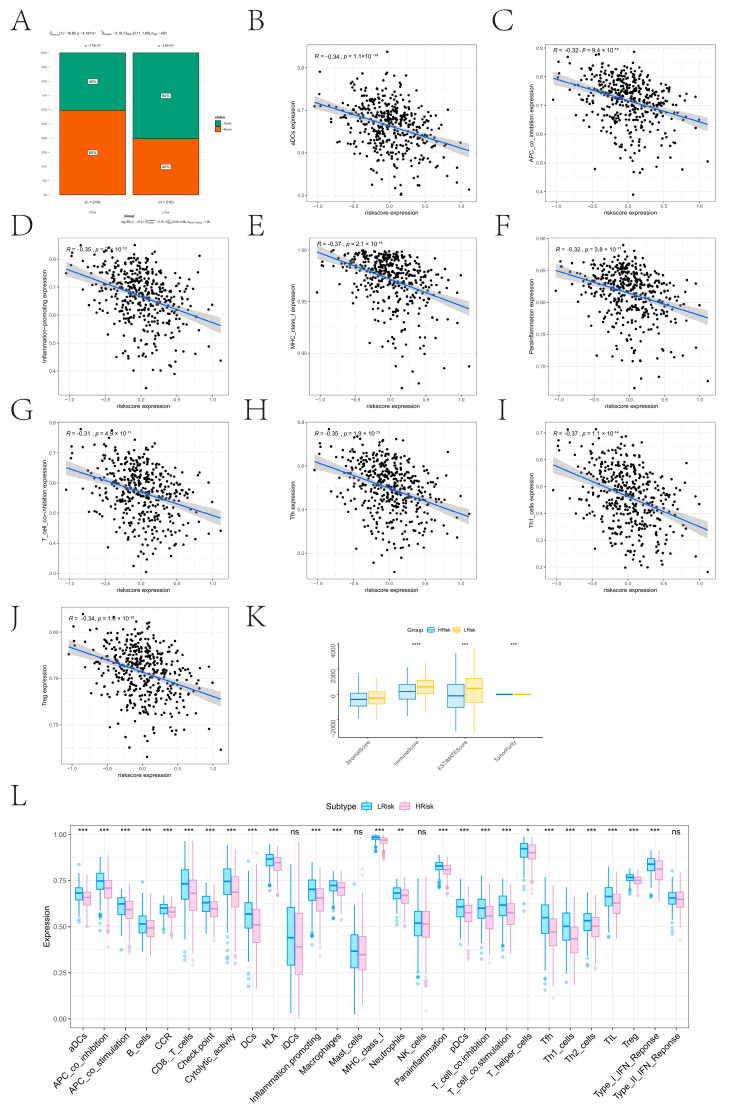
Risk score association with immune infiltration and immunotherapy response. (**A**) TIDE algorithm-based prediction of immunotherapy response differences between high- and low-risk groups. Statistical differences were evaluated using the Wilcoxon rank-sum test. (**B**–**J**) Correlation of risk scores with specific immune cell populations and immune-related functional signatures. Correlations were assessed using Spearman correlation analysis. (**K**) Comparative analysis of ESTIMATE-derived stromal, immune, and overall scores, along with tumor purity between risk groups. Statistical significance was assessed using the Wilcoxon rank-sum test. (**L**) Differences in immune cell subsets and immune functions between high- and low-risk categories. A total of 429 TCGA samples were included in the analysis. “ns” indicates *p* > 0.05, “*” indicates *p* < 0.05, “**” indicates *p* < 0.01, “***” indicates *p* < 0.001, and “****” indicates *p* < 0.0001.

Computational inference indicated reduced immunotherapy responsiveness in high-risk patients (Figure 7A). Risk scores inversely correlated with multiple immune-related gene signatures (Figure 7B–J). Additionally, ESTIMATE analyses demonstrated substantial differences in immune ESTIMATE scores and tumor purity between the high- and low-risk groups (Figure 7K). In addition, compared with ESTIMATE-derived immune infiltration and tumor purity indicators, the risk score showed better predictive performance for 5-year OS, suggesting that its prognostic value may not be fully explained by global immune infiltration or tumor purity alone (Figure S7). Differences were significant for several immune subtypes, including type I interferon response, Tregs, tumor-infiltrating lymphocytes (TILs), Th1 cells, Th2 cells, T-helper cells, T-cell co-stimulation, Tfh, cytolytic activity, CD8+ T cells, chemokine receptor (CCR) expression, antigen-presenting cell (APC) co-stimulation, and B cells (Figure 7L).

### 3.10. Independent Prognostic Value and Nomogram Construction

**Figure 8:**
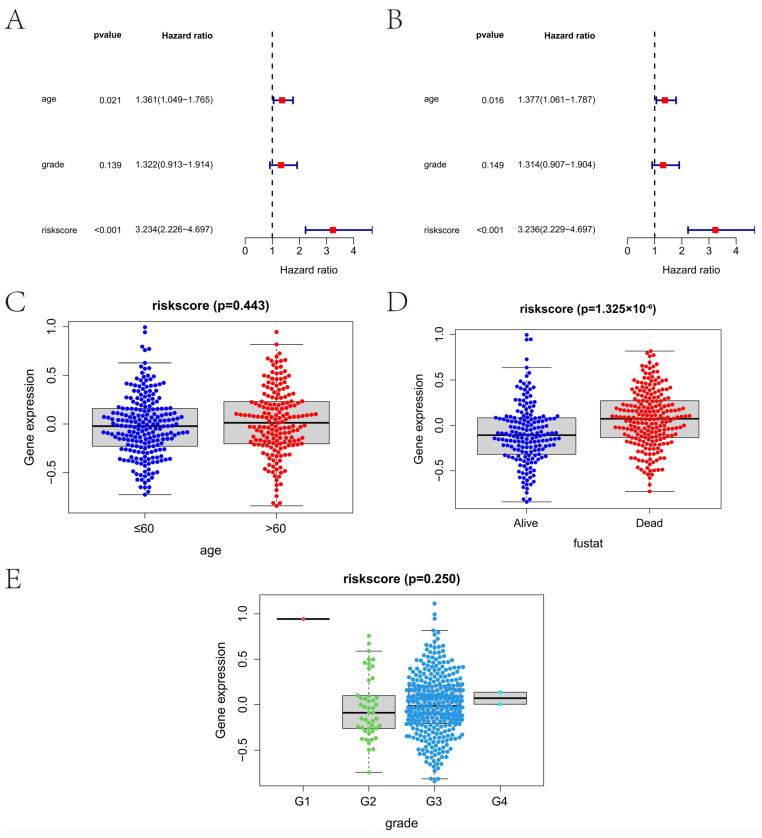
Independent prognostic value of the risk score and correlation with clinical features. (**A**,**B**) Univariate and multivariate Cox regression analyses affirming the independent prognostic relevance of the risk score. Hazard ratios and 95% confidence intervals were calculated using Cox proportional hazards models, and statistical significance was assessed using Wald tests. (**C**) Association between risk score and patient age, evaluated using the Wilcoxon rank-sum test. (**D**) Distribution of risk score according to overall survival status, assessed using the Wilcoxon rank-sum test. (**E**) Association between risk score and tumor grade, evaluated using the Kruskal–Wallis test. A total of 429 TCGA patients with OC were included in the analysis.

Both univariate and multivariate Cox regression analyses supported the independent prognostic significance of the risk score for OS in OC (Figure 8A,B). Additionally, risk scores were significantly associated with patient survival status (*p* < 0.05), while no significant differences were observed with respect to age or tumor grade (Figure 8C–E).

**Figure 9:**
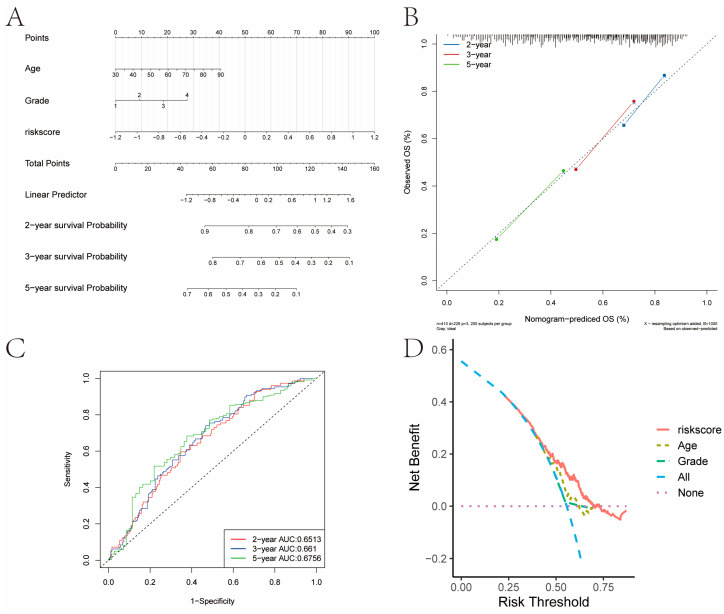
Construction and validation of the prognostic nomogram. (**A**) Nomogram integrating age, tumor grade, and risk score for predicting 2-, 3-, and 5-year OS. The model was constructed based on multivariate Cox regression analysis. (**B**) Calibration plots illustrating concordance between nomogram-predicted and observed OS rates at designated time points. (**C**,**D**) ROC and DCA assessing the predictive accuracy and clinical utility of the nomogram. A total of 429 TCGA OC patients were included in the analysis.

To explore potential clinical utility, a comprehensive nomogram incorporating risk scores alongside relevant clinical features was constructed (Figure 9A). Calibration plots revealed strong agreement between nomogram predictions and observed survival outcomes at 2-, 3-, and 5-year time points (Figure 9B). Further assessments of predictive performance and potential clinical applicability were performed using time-dependent ROC curves and decision curve analyses (DCA) (Figure 9C,D).

### 3.11. Drug Sensitivity Prediction and Pathway Enrichment

In the context of potential therapeutic implications, drug sensitivity was in silico predicted using the oncoPredict R package based on the GDSC database. The analysis suggested that high-risk patients may exhibit increased sensitivity to Alpelisib and Linsitinib, whereas low-risk patients may be more sensitive to Ruxolitinib, LCL161, IWP-2, and LGK974 (Figure S8A). GSVA revealed enrichment of several tumor-associated signaling pathways within the high-risk group, notably Wnt/β-catenin signaling, Hedgehog signaling, apical surface interactions, and Kras signaling pathways (Figure S8B). GSEA further highlighted significant enrichment of taste transduction, protein export, and proteasome pathways (Figure S8C).

## 4. Discussion

OC is a highly heterogeneous gynecological malignancy, and conventional Tumor-Node-Metastasis (TNM) staging is insufficient for personalized diagnosis and treatment [39]. scRNA-seq provides a powerful tool for dissecting cellular diversity and underlying molecular mechanisms in OC [40]. In the current study, integrated single-cell and bulk transcriptomic analyses identified key B cell and plasma cell populations within the TME. Subsequently, a prognostic risk-scoring model was developed, and the core gene *FUT8* was further investigated in functional analyses. A nomogram was further established as an exploratory tool for survival estimation in patients with OC. These findings provide new insights into the molecular characteristics of OC and may inform future studies on prognostic stratification and therapeutic target discovery.

The prognostic model, developed from marker genes associated with B cell and plasma cell populations, showed moderate predictive accuracy and achieved statistically significant survival separation between the high- and low-risk groups. Although the model outperformed traditional prognostic indicators based solely on immune cell infiltration abundance, it did not reach the accuracy required for use as a standalone clinical decision-making tool. Historically, research on OC immune microenvironments has predominantly centered on T cells; however, emerging studies underscore the pivotal contributions of B cells and plasma cells to antitumor immunity [22]. The intricate interactions among immune cells within the TME significantly affect therapeutic responses [23]. Notably, most genes incorporated into our prognostic model demonstrated negative regression coefficients, collectively suggesting protective roles. Genes such as X-box binding protein-1 (*XBP1*), protein disulfide isomerase A4 (*PDIA4*), and mesencephalic astrocyte-derived neurotrophic factor (*MANF*) are notably involved in ER stress responses and the unfolded protein response (UPR), critical processes underlying antibody synthesis and secretion in plasma cells [41,42,43]. Consequently, our prognostic model may reflect not only the abundance of B and plasma cells but also their functional diversity within the TME. Recent advances in scRNA-seq have facilitated such precise functional characterizations, underscoring its substantial potential for precise OC classification, prognostication, and treatment selection.

Within our prognostic signature, *FUT8* emerged as a key gene. Consistent with prior studies, *FUT8* expression was elevated in OC tissues and cell lines [44,45]. Nonetheless, higher *FUT8* levels correlated positively with patient survival outcomes, and experimental overexpression of *FUT8* suppressed malignant characteristics in vitro, although further validation through loss-of-function studies remains essential to establish definitive causation. This apparent contradiction implies a context-dependent functional role for *FUT8* in OC. Multiple mechanisms could explain this divergence, potentially reflecting differential substrate availability and specificity of *FUT8*-mediated glycosylation across various tissues and cellular contexts. For instance, in non-small cell lung cancer, *FUT8* facilitates tumor progression via core fucosylation of epidermal growth factor receptor (*EGFR*) specifically within cancer-associated fibroblasts [46]. In breast cancer, *FUT8* enhances EMT and invasion through modification of transforming growth factor beta (*TGF-β*) receptors [47]. These findings suggest that downstream signaling outcomes may depend on cellular context rather than reflecting a uniform mechanism. Second, the net effect of *FUT8* may differ between tumor-cell-intrinsic regulation and TME-mediated processes, particularly immune and stromal interactions. The protective prognostic association observed in our cohort may reflect both direct tumor-suppressive effects and a B cell/plasma cell-associated immune microenvironment, whereas the pro-tumorigenic effects reported in other studies may be driven by macrophage-mediated immune modulation [45]. Third, OC is highly heterogeneous, and differences in molecular subtype and TME composition, such as HGSOC versus low-grade serous ovarian carcinoma (LGSOC) or distinct mutational landscapes, may further influence *FUT8* function. These variables are not mutually exclusive and may collectively shape the biological role of *FUT8*. Consistent with this interpretation, our single-cell analysis showed that *FUT8* was broadly expressed across multiple cell types within the OC TME, with relatively higher expression in B cells and plasma cells. This suggests that its prognostic value may reflect combined activity in tumor and immune compartments. This pan-cellular expression pattern is consistent with the universal biological function of *FUT8* as the only enzyme responsible for core fucosylation of N-linked glycoproteins [48]. In addition, previous studies have reported tumor-promoting roles of *FUT8* across different cancer types [47,49,50], suggesting that *FUT8* may exert oncogenic functions in specific tumor contexts and further supporting its context-dependent and cancer-type-specific functional variability. The downstream mechanisms underlying the potential role of *FUT8* were explored using transcriptome sequencing in A2780 cells after *FUT8* overexpression, coupled with virtual knockout analyses for bidirectional validation. *FUT8* overexpression significantly enriched glycosylation-related pathways, particularly N-glycan biosynthesis, as well as immune-related GO terms. Virtual knockout analyses identified pathways related to hexose metabolism and N-linked glycosylation of proteins. Collectively, these findings indicate that FUT8 may be involved in N-glycosylation-dependent signaling pathways and may be associated with cellular processes such as apoptosis and cell cycle regulation. Additionally, GSEA indicated enrichment of ECM–receptor interaction pathways, suggesting *FUT8* may alter adhesion molecule glycosylation, potentially influencing intercellular communication within the TME. However, because these findings are derived from pathway enrichment analyses, the direct causal relationship between *FUT8*-mediated glycosylation and activation of these pathways requires further experimental validation.

Patient stratification using risk scores identified distinct immune-infiltration phenotypes within the high-risk subgroup, characterized by reduced populations of crucial antitumor immune cells. CD8+ T cells, as primary cytotoxic effectors, directly mediate tumor cell killing within the TME [51], and their abundance positively correlates with improved prognosis in OC [52]. The simultaneous reduction of Tfh, B cells, and CD8+ T cells suggests a coordinated pattern of immune cell co-depletion involving these subsets. Specifically, Tfh cells facilitate B cell maturation into antibody-secreting plasma cells and support CD8+ T cell activation, proliferation, and memory formation [53,54]. The decreased presence of Tfh cells in high-risk patients likely reflects impaired humoral and cellular immunity, indicative of a more immunosuppressed TME. Correlation analyses further confirmed significant associations between risk scores and multiple immune infiltration characteristics, reinforcing the model’s capacity to represent a comprehensive immune microenvironment profile.

GSVA, performed based on prognostic risk stratification, indicated enrichment of several tumor-associated signaling pathways, including Kras and apical surface, in the high-risk group. The Wnt/β-catenin pathway influences OC tumorigenesis, progression, chemoresistance, and prognosis [55], with its pathway components recognized as biomarkers and therapeutic targets associated with advanced tumor stages [56,57]. Similarly, Kras signaling, frequently activated in LGSOC [58], promotes invasiveness [59] and chemoresistance in OC [60]. Hedgehog signaling likewise plays essential roles in OC progression, invasion, and chemotherapy resistance [61], frequently cooperating with Wnt and Kras pathways [62,63]. Thus, *FUT8*-associated N-glycosylation may influence receptor-mediated signaling processes involved in these pathways, although direct mechanistic evidence remains to be established.

In silico drug sensitivity analysis based on prognostic risk scores predicted differential responsiveness between high- and low-risk groups. High-risk patients exhibited enhanced predicted sensitivity to Alpelisib and Linsitinib, potentially indicative of distinct molecular vulnerabilities. Conversely, low-risk patients demonstrated higher predicted sensitivity to Ruxolitinib, LCL161, IWP-2, and LGK974. Alpelisib has demonstrated effectiveness in molecularly defined OC subsets [64], while Linsitinib inhibits insulin-like growth factor-1 (IGF-1) signaling [65]. However, these computational predictions require experimental confirmation and clinical validation.

**Figure 10:**
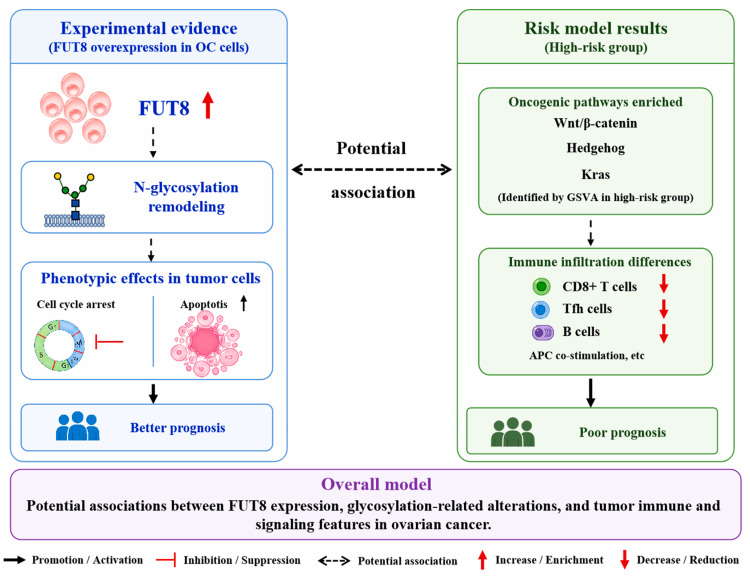
Proposed model of potential *FUT8*-associated regulatory networks in OC. *FUT8* may be associated with alterations in N-glycan core fucosylation and signaling pathways, including Wnt/β-catenin, Hedgehog, and Kras signaling. *FUT8* expression is associated with cell cycle arrest, increased apoptosis, a B/plasma cell-enriched immune microenvironment, and improved prognosis in OC. The immune-related findings are correlative and do not indicate direct regulatory effects of *FUT8* on immune cells. Green arrows indicate activation or enrichment, red lines indicate inhibition or suppression, and dashed lines indicate hypothetical associations requiring further experimental validation.

Figure 10 presents a conceptual framework summarizing potential interactions between *FUT8*-related N-glycosylation remodeling and key oncogenic pathways in OC. Elevated *FUT8* expression may influence signaling networks involving the Wnt/β-catenin, Hedgehog, and Kras pathways via glycosylation-dependent mechanisms. Moreover, increased *FUT8* levels may correlate with enriched B cell/plasma cell immune microenvironments and improved clinical outcomes in OC.

This study has several limitations. First, the single-cell analysis included a limited number of samples derived from publicly available datasets, without validation in an independent, multicenter single-cell cohort. Second, potential batch effects may exist between TCGA and GTEx datasets, and several important clinical variables, such as FIGO stage, BRCA/HRD status, and treatment response, were not fully included because of data limitations, which may affect model interpretability. Third, although repeated random resampling was used during exploratory model construction, the final 13-gene signature was not selected based on formal gene-level selection frequencies across all resampling iterations. Therefore, the stability and generalizability of this signature require further confirmation using predefined bootstrap or repeated cross-validation frameworks and independent prospective cohorts. Fourth, immunotherapy response and drug sensitivity analyses were based on computational predictions and require further investigation. Fifth, the independent prognostic value of the risk score after formal multivariate adjustment for immune score and tumor purity has not been explicitly assessed, and this issue should be addressed in future studies. Sixth, functional validation of *FUT8* was limited to gain-of-function experiments without loss-of-function or rescue assays; therefore, causal inference remains incomplete. In addition, the proposed N-glycosylation-related mechanisms lack direct glycoproteomic or substrate-level validation. Finally, OC is highly heterogeneous, and the datasets used in this study were mainly enriched for HGSOC. Therefore, the findings are most applicable to HGSOC rather than all OC subtypes, and the cell lines used do not fully capture tumor heterogeneity. Future studies should validate these findings in independent prospective cohorts, systematically identify *FUT8* glycoprotein substrates, and integrate spatial and functional multi-omics approaches to better clarify its role in the TME.

## 5. Conclusions

In summary, this study characterized B cell and plasma cell populations at single-cell resolution, developed an exploratory and biologically relevant prognostic risk model, and identified *FUT8* as a potential tumor-suppressive regulator in OC. These findings provide a framework for improved prognostic stratification and suggest that *FUT8*-related glycosylation alterations may have potential biomarker value in OC. Additional prospective studies in clinical cohorts and rigorous in vivo validations are necessary to confirm *FUT8*’s clinical applicability.

## Data Availability

The RNA-seq raw datasets for this study can be found in the National Center for Biotechnology Information (NCBI) Sequence Read Archive (SRA), with accession numbers PRJNA1457258 and PRJNA1456515. Raw sequencing data from public repositories can be obtained from TCGA (https://cancergenome.nih.gov, accessed on 14 February 2025) and GEO (https://www.ncbi.nlm.nih.gov/geo, accessed on 14 February 2025). The original contributions presented in the study are included in the article/Supplementary Materials. Further inquiries can be directed to the corresponding author.

## Supplementary Materials

The following supporting information can be downloaded at: https://www.mdpi.com/article/10.3390/genes17070784/s1, Figure S1: Preliminary single-cell data processing; Figure S2: Pseudotime trajectory analysis of B and plasma cells; Figure S3: SCENIC analysis of transcriptional regulatory networks in B and plasma cells; Figure S4: Activity variations in immune–metabolic pathways; Figure S5: Effect of *FUT8* overexpression on proliferation and apoptosis gene expression; Figure S6: Transcriptomic analysis associated with in silico *FUT8* perturbation; Figure S7: Comparison of 5-year OS predictive performance between the risk score and ESTIMATE-derived indicators; Figure S8: Drug sensitivity and pathway enrichment analyses by risk score; Table S1: Number of cells retained per sample after quality control; Table S2: Differentially expressed mRNAs; Table S3: Functional analysis of DEGs; Table S4: GSEA; Table S5: Primers used for qRT-PCR.

## Abbreviations

The following abbreviations are used in this manuscript:

AUC	area under the curve
CA125	serum cancer antigen 125
CCK-8	cell counting kit-8
CCR	chemokine receptor
CIs	confidence intervals
CTLs	cytotoxic T lymphocytes
DCA	decision curve analyses
DEGs	differentially expressed genes
ECM	extracellular matrix
EGFR	epidermal growth factor receptor
FIGO	International Federation of Gynecology and Obstetrics
FUT8	fucosyltransferase 8
GDF15	growth differentiation factor 15
GDSC	Genomics of Drug Sensitivity in Cancer
GEO	Gene Expression Omnibus
GO	Gene Ontology
GSEA	Gene Set Enrichment Analysis
GSVA	Gene Set Variation Analysis
GTEx	Genotype–Tissue Expression
HE4	human epididymis protein 4
HGSOC	high-grade serous ovarian carcinoma
HOXA1	homeobox A1
HRs	Hazard ratios
IC_50_	half-maximal inhibitory concentration
IGF-1	insulin-like growth factor-1
IHC	immunohistochemistry
KEGG	Kyoto Encyclopedia of Genes and Genomes
LGSOC	low-grade serous ovarian carcinoma
MAD	median absolute deviation
MANF	mesencephalic astrocyte-derived neurotrophic factor
OC	ovarian cancer
OS	overall survival
PCA	principal component analysis
PDIA4	protein disulfide isomerase A4
qRT-PCR	quantitative real-time polymerase chain reaction
ROC	receiver operating characteristic
ScRNA-seq	single-cell RNA sequencing
SCENIC	single-cell regulatory network inference and clustering
SEM	standard error of the mean
ssGSEA	single-sample gene set enrichment analysis
TCGA	The Cancer Genome Atlas
TF	transcription factors
Tfh	follicular helper T cells
TILs	tumor-infiltrating lymphocytes
TLS	tertiary lymphoid structures
TME	tumor microenvironment
TGF-β	transforming growth factor beta
Tregs	regulatory T cells
TNM	Tumor-Node-Metastasis staging system
UMAP	Uniform Manifold Approximation and Projection
UPR	unfolded protein response
WB	Western blotting
XBP1	X-box binding protein 1
