# Supplementary material for: Integrated Single-Cell and Bulk Transcriptomic Analyses Identify a B Cell- and Plasma Cell-Associated Prognostic Signature and a Candidate Tumor-Suppressive Role for FUT8 in Ovarian Cancer
Source: Genes (Basel). 2026 Jul 8;17(7):784. doi: 10.3390/genes17070784 (PMC13410260; doi:10.3390/genes17070784)
Supplement: Supplementary file 1 [file genes-17-00784-s001.zip › supplementary materials.pdf]

1. Materials and Methods

1.1 Data download

We obtained the processed raw expression data for ovarian cancer from the TCGA database, which comprised expression profiles of 429 patients. A total of 88 normal samples were obtained from the Genotype-Tissue Expression (GTEx) database. These samples were combined with TCGA data and normalized for differential expression analysis.

1.2 Survival analysis

Survival analysis refers to a group of statistical techniques designed to manage time-to-event data. It is extensively used in disciplines like medicine, biology, and epidemiology to assess how different factors affect survival time. The purpose of this study is to apply survival analysis techniques to investigate how gene expression levels affect the overall survival of patients with ovarian cancer.

1.3 Cell culture

The study employed IOSE-80, A2780, and SKOV-3 cell lines to measure *FUT8* expression level and to perform gain-of-function experiment in vitro. These lines were acquired from iCell Bioscience, Shanghai, China. IOSE-80 cells are immortalized human ovarian surface epithelial cells derived from normal ovarian epithelium and are widely used as a non-tumorigenic epithelial control model in ovarian cancer research. These cells are immortalized by SV40 large T antigen. The IOSE-80 and SKOV-3 cell lines were grown in RPMI 1640 medium (HyClone, Cytiva, Logan, UT, USA) containing 10% fetal bovine serum (FBS, Gibco, Thermo Fisher Scientific, Waltham, MA, USA) and 1% penicillin/streptomycin, while the A2780 cell line was maintained in DMEM (Gibco, Thermo Fisher Scientific, Waltham, MA, USA) with 10% FBS and 1% penicillin/streptomycin. The cell cultures were subjected to incubation at 37°C in an atmosphere that was humidified with 5% CO_2.

1.4 Immunohistochemical staining

Tissue microarray (TMA) (Cat No. YP-FOV8011a) paraffin blocks of OC tissues were purchased from Shanghai Yblbio (Shanghai, China). A total of 80 pairs of cancerous and paracancerous tissue samples were subjected to IHC staining. Each TMA slide was first stained with a mouse anti-FUT8 antibody (dilution, 1:700; 66118-1-Ig; Proteintech, Wuhan, China), and then incubated with horseradish peroxidase-conjugated goat anti-mouse immunoglobulin G antibody (dilution, 1:50; A0216; Beyotime; Shanghai, China). After rinsing, color was developed using 3, 3′-diaminobenzidine (DAB, Servicebio, Inc.). Sections were counterstained with hematoxylin and photographed at 400×magnification using an XSP-C204 microscope (Chongqing Optec Instrument Co., Ltd. (COIC), Chongqing, China). Images were then captured using Pannoramic viewer (3DHISTECH Kft; Budapest, Hungary) and analyzed using HALO. Immunohistochemistry score (H-SCORE) was calculated as H-SCORE=$\sum(PI \times I)$ = (percentage of cells with weak intensity × 1) + (percentage of cells with moderate intensity× 2) + (percentage of cells with strong intensity × 3), where PI is the proportion of positive cells among all cells in the section and I is the coloration intensity. Cancerous tissue samples were divided into low and high expression groups according to H-score, and compared with paracancerous tissue samples, respectively.

1.5 Western blot

The total protein was isolated from cells using Cell lysis buffer for Western and IP (Beyotime Biotechnology, Shanghai, China) and the protein concentration was determined using the Enhanced BCA Protein Assay Kit (Beyotime Biotechnology, Shanghai, China). The FUT8 polyclonal antibody was purchased from Proteintech, Inc. The GAPDH monoclonal antibody was purchased from Beyotime, Inc. Equal amounts of protein were separated by 10% sodium dodecyl sulfate gel electrophoresis under denaturing and non-reducing conditions and then transferred to polyvinylidene fluoride (PVDF) membranes. The PVDF membranes were blocked, then incubated (1 h, 37°C) with the FUT8 antibody (dilution, 1:2,000; 66118-1-Ig; Proteintech, Wuhan, China) and GAPDH antibody (dilution, 1:1,000; AF1186; Beyotime; Shanghai, China) which diluted in Primary Antibody Dilution Buffer (Beyotime). After washing in phosphate-buffered saline with Tween 20, the blots were incubated with horseradishperoxidase-conjugated goat anti-mouse/rabbit immunoglobulin G antibody (dilution, 1:2,000; A-216/A0208; Beyotime; Shanghai, China) which diluted in Secondary Antibody Dilution Buffer (Beyotime Biotechnology, Shanghai, China). The signals were visualized using a BeyoECL Star kit (Beyotime Biotechnology, Shanghai, China).

1.6 Transfection

Upon reaching a cellular confluence of 70%, the cells underwent transfection with pcDNA3.1 vector and pcDNA3.1-FUT8 vector utilizing Lipofectamine 2000 reagent (Invitrogen, Thermo Fisher Scientific, Carlsbad, CA, USA). The cells were utilized for subsequent experiments 48 h post-transfection.

1.7 RNA extraction and qRT-PCR

The extraction of total RNA from cell lines was performed by utilizing the TRIzol reagent (Invitrogen, Carlsbad, CA, USA) following the directions provided by the manufacturer. The reverse transcription of total RNA (1μg) was conducted employing a FastKing gDNA Dispelling RT SuperMix (Tiangen, Beijing, China). The qPCR procedure was conducted employing Talent qPCR PreMix (Tiangen, Beijing, China) following the manufacturer's rules. The primers were showed in Table S5. The internal control *GAPDH* was utilized to normalize all amplifications. All samples were assayed in triplicate, and all data were examined employing the $2^{-\triangle\triangle Ct}$ technique.

1.8 CCK-8 assay

The experiment involved seeding cells in 96-well plates and subsequent transfection with pcDNA3.1 vector and pcDNA3.1-FUT8 vector. Following transfection, a 10 μL solution of CCK-8 (Beyotime, Shanghai, China) was introduced for each well to assess cell viability at 0, 24, 48, and 72 h. The detection of absorbance was conducted utilizing a FilterMax F5 plate reader (Molecular Devices, Sunnyvale, CA, USA) set at a wavelength of 450 nm.

1.9 Flow cytometry

The study involved the seeding of cells in 6-well plates and subsequent transfection with pcDNA3.1 and pcDNA3.1-FUT8 for 48h. The cells were harvested and subsequently resuspended in 75% ethanol for an overnight duration. The cells were stained using the Cell Cycle Analysis Kit (Beyotime, Shanghai, China) following the manufacturer's rules. The cell cycle was quantified using SA3800 flow cytometer (Sony Biotechnology, Tokyo, Japan) and Modfit LT 5.0 (version 5.0, Verity Software House, Topsham, ME, USA). The

cells collected underwent staining employing an Annexin V-FITC apoptosis detection kit (Beyotime Biotechnology, Shanghai, China) and were subsequently analyzed through a flow cytometer (Sony Biotechnology, Tokyo, Japan) following the manufacturer's directions. The trials were conducted separately and in triplicate.

1.10 Transcriptome sequencing

The A2780 cells were transfected with pcDNA3.1 vector and pcDNA3.1-FUT8 vector, and were utilized for transcriptome sequencing 48 h post-transfection. Each experimental condition included three independent biological replicates (n=3). Transcriptome sequencing was commissioned to Guangdong OmicsMaster Biotechnology Co., Ltd. Briefly, mRNA was enriched from total RNA using oligo(dT) magnetic beads, followed by purification and thermal fragmentation. First-strand and second-strand cDNA were synthesized sequentially, then subjected to end repair, A-tailing, adapter ligation, and fragment size selection. After PCR amplification, libraries were sequenced on the Illumina NovaSeq X Plus with paired-end 150 bp strategy. Raw reads were quality-controlled using fastp (version 0.18.0): adapter-containing reads, reads with >10% unknown bases (N), and low-quality reads with >50% bases of Q-value ≤ 20 were removed. Ribosomal RNA (rRNA) was depleted by alignment with Bowtie2 (version 2.2.8), and the remaining clean reads were mapped to the human reference genome via HISAT2 (version 2.1.0) with default parameters. Gene transcript assembly and expression quantification were performed using StringTie (version 1.3.1) combined with RSEM software, and both raw read count matrix and TPM (Transcripts Per Kilobase of exon model per Million mapped reads) matrix were generated; raw counts were used as input for differential expression analysis, while TPM values were applied for gene expression level visualization and cross-sample comparison. Differential expression analysis was performed using raw read count data within the DESeq2 framework, which applies a negative binomial statistical model and built-in size factor normalization to account for library size differences. Significantly differentially expressed genes were screened with the criteria of FDR<0.05 and |log2FC|>1. Eight differential genes were selected for qRT-PCR validation. GO and KEGG pathway enrichment analyses were performed on DEGs using hypergeometric test, with Benjamini-Hochberg FDR correction applied and FDR ≤ 0.05 set as the significance threshold. Given the limited number of DEGs, all enrichment results were interpreted as exploratory functional associations rather than definitive pathway activation conclusions. GSEA was conducted on the full gene expression matrix using the MSigDB database (version 7.0) reference gene sets, with Signal2Noise ranking metric and default parameters.

1.11 virtual knockout

The scTenifoldKnk algorithm was used to perform "virtual knockout" experiments on the *FUT8* gene to evaluate the core role of the FUT8 gene in the single-cell transcriptome regulatory network. The key assumption of this algorithm is that the topological structure of the gene regulatory network (GRN) implies the functional dependence between genes, and the deletion of a single gene will disrupt the network homeostasis. The experiment only requires wild-type single-cell transcriptome data to construct a gene regulatory network. Genes were ranked according to log2 fold-change derived from the virtual knockout analysis. This ranked gene list was used for GSEA. GO enrichment

analysis was also performed to identify significantly enriched biological processes associated with *FUT8* perturbation.

2. Figures

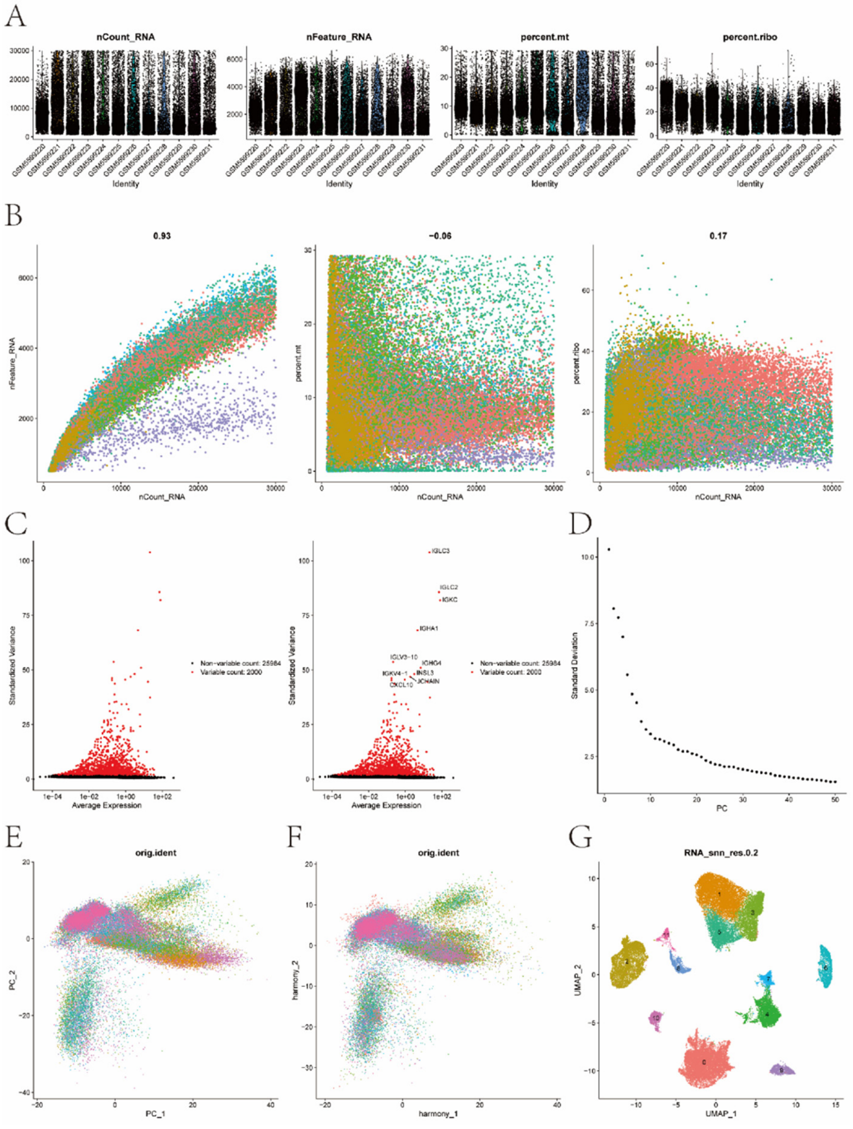

Figure S1. Preliminary single-cell data processing. (**A**) Quality control metrics per sample. (**B**) Correlation between sequencing depth, mitochondrial content, and RNA counts per cell; scatter plots show mitochondrial gene expression relative to RNA counts. (**C**) Identification and variance analysis of significantly different genes between cells. (**D**) Variance ranking plot for each PC. (**E, F**) PCA illustrating PC distribution; cells are color-coded by sample. (**G**) Classification of cells into 12 clusters by UMAP based on significant PCA components (resolution = 0.2). A total of 46,235 single cells from 12 samples (7 tumor and 5 normal ovarian tissues) were analyzed.

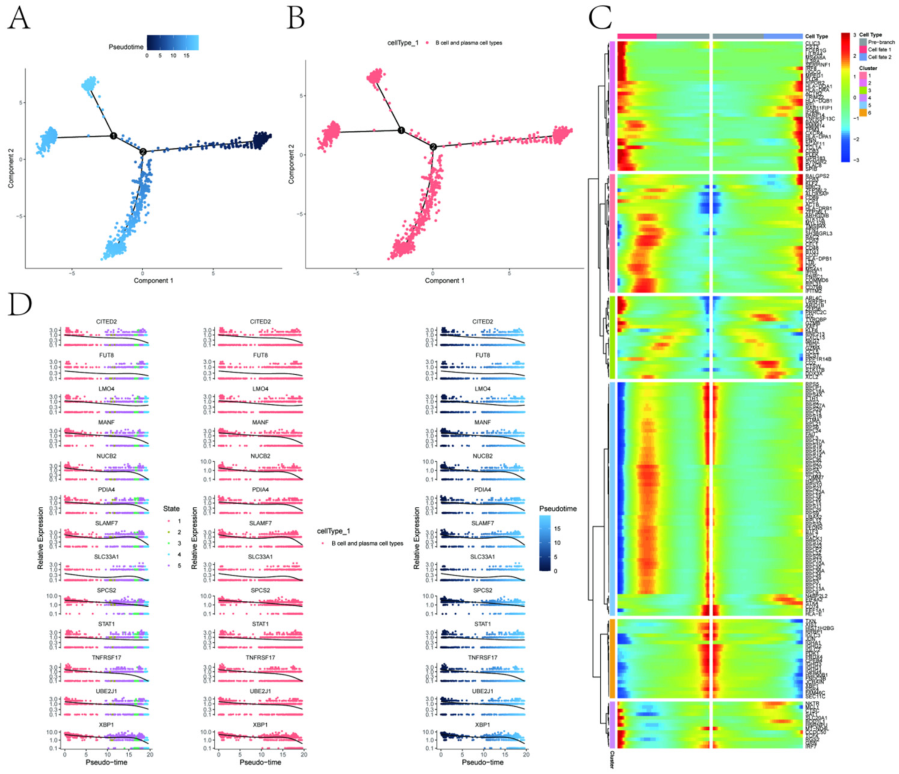

Figure S2. Pseudotime trajectory analysis of B and plasma cells. (**A, B**) Monocle pseudotime trajectories colored by pseudotime (A) and cell type (B). (**C**) Heatmap of dynamic gene expression along developmental trajectory. (**D**) Expression patterns of prognostic model genes over pseudotime.

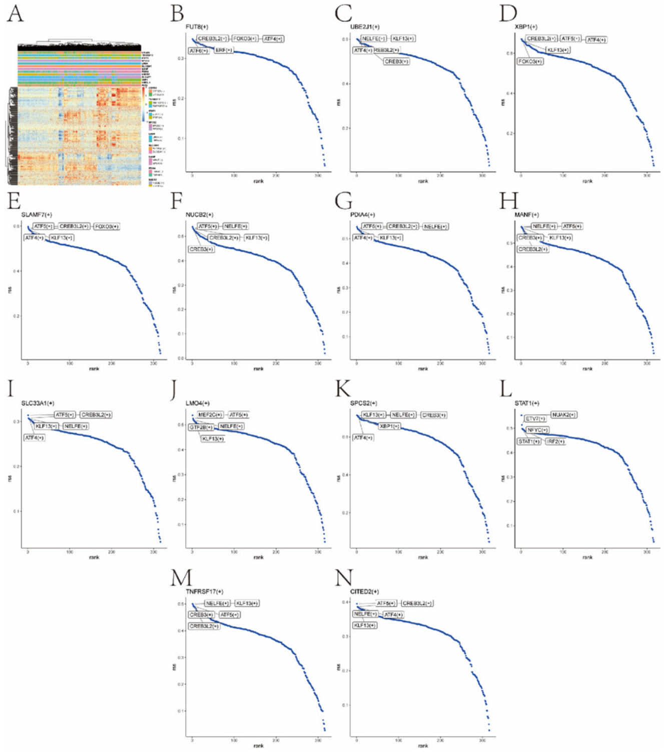

Figure S3. SCENIC analysis of transcriptional regulatory networks in B and plasma cells. (**A**) SCENIC analysis illustrating transcription factor regulons. (**B-N**) Regulon activity ranking plots for 12 prognostic transcription factors, highlighting top target genes.

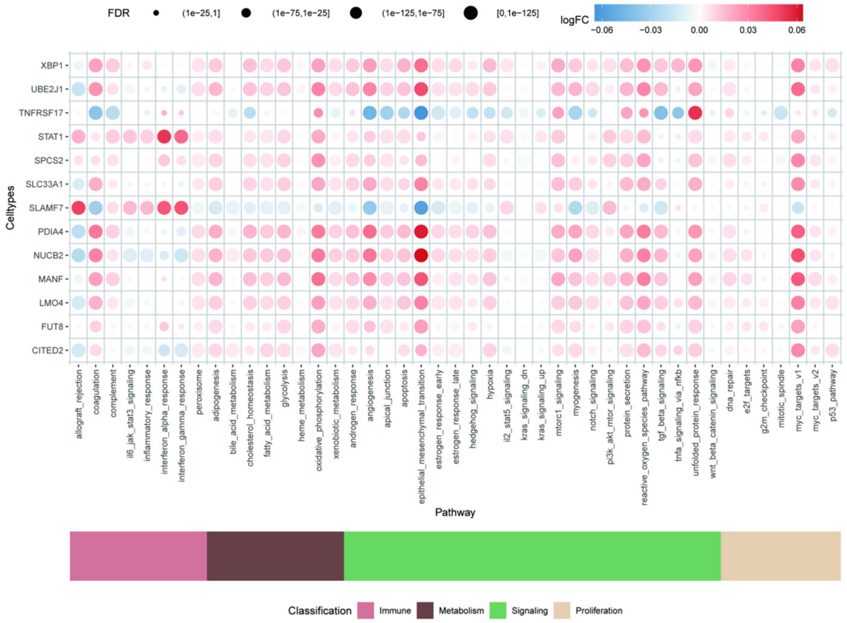

Figure S4. Association between prognostic signature genes and immune- and metabolism-related pathway activity inferred by AUCell analysis. Bubble plot showing the activity scores of curated pathways across 13 signature genes in single-cell transcriptomic data. Dot size indicates statistical significance (FDR-adjusted P values), and color represents normalized pathway activity scores. A total of 13 signature genes and curated pathway gene sets were included in the analysis.

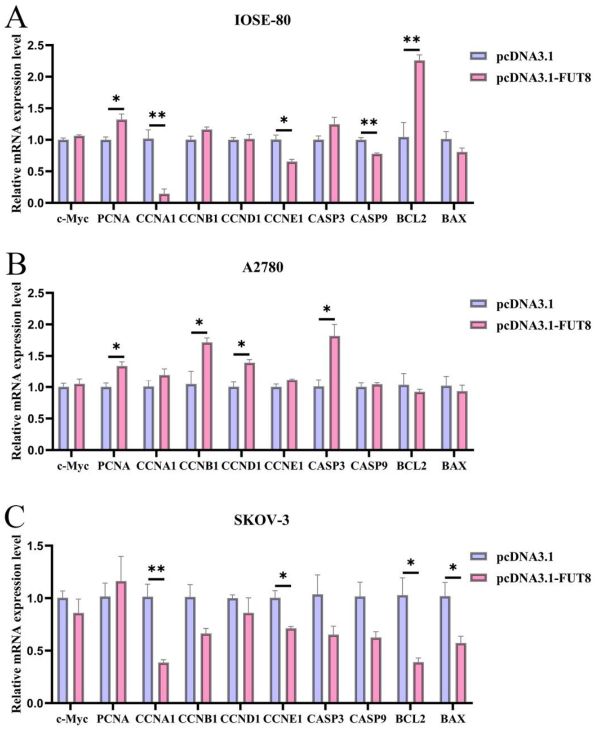

Figure S5. Effect of *FUT8* overexpression on cell cycle- and apoptosis-related gene expression (n = 3). (**A**), A2780 (**B**), and SKOV-3 (**C**) cells transfected with pcDNA3.1 or pcDNA3.1-*FUT8*. Statistical significance was assessed using t-test. Data are presented as mean ± SEM. "*" indicates $P < 0.05$, "**" indicates $P < 0.01$.

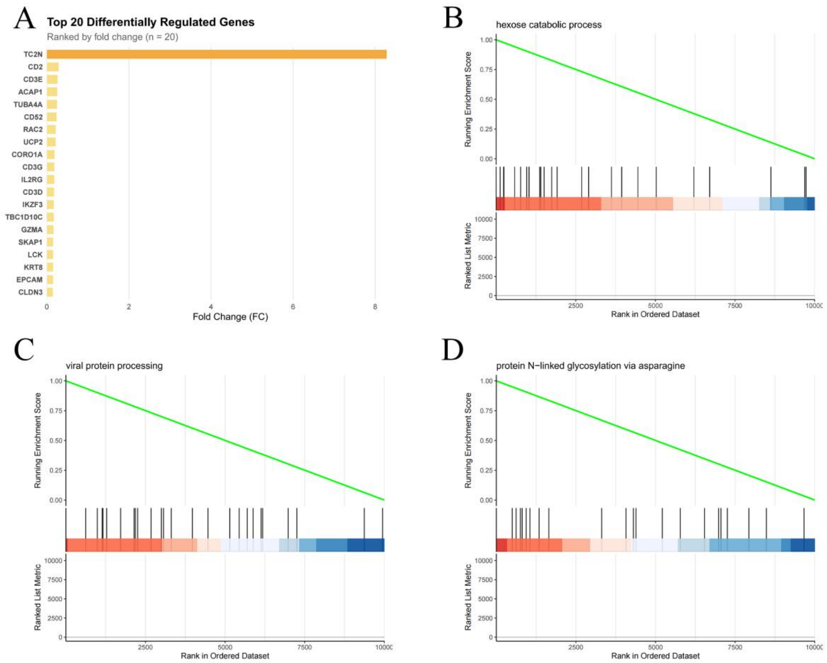

Figure S6. Transcriptomic analysis associated with in silico *FUT8* perturbation. (**A**) Top 20 differentially expressed genes identified following *FUT8* in silico perturbation. (**B-D**) GSEA illustrating key pathways downregulated following *FUT8* virtual knockout.

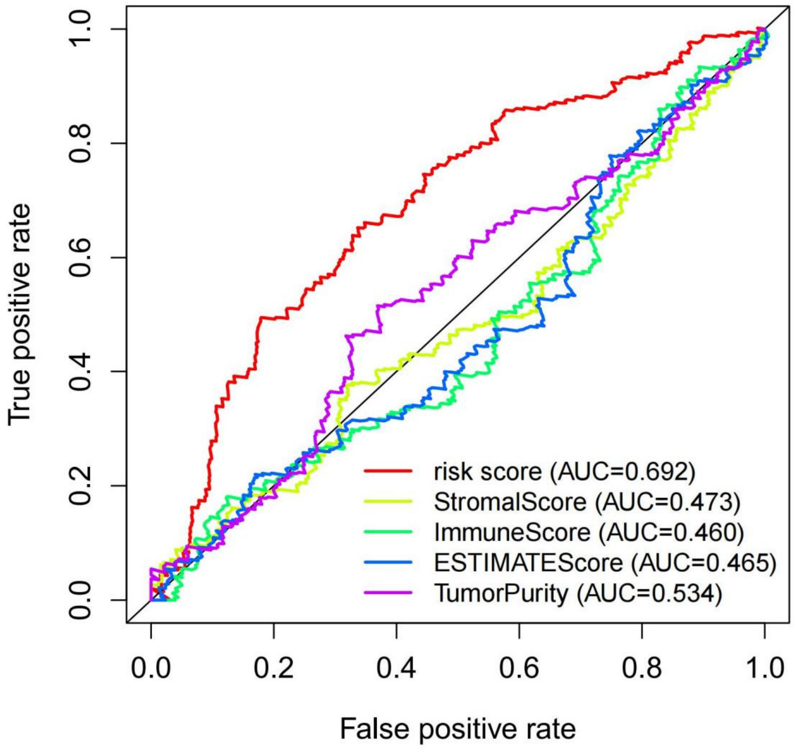

Figure S7. Comparison of 5-year OS predictive performance between the risk score and ESTIMATE-derived indicators. ROC curves show the prognostic performance of the risk score, StromalScore, ImmuneScore, ESTIMATEScore, and TumorPurity for 5-year OS. The corresponding AUC values are indicated.

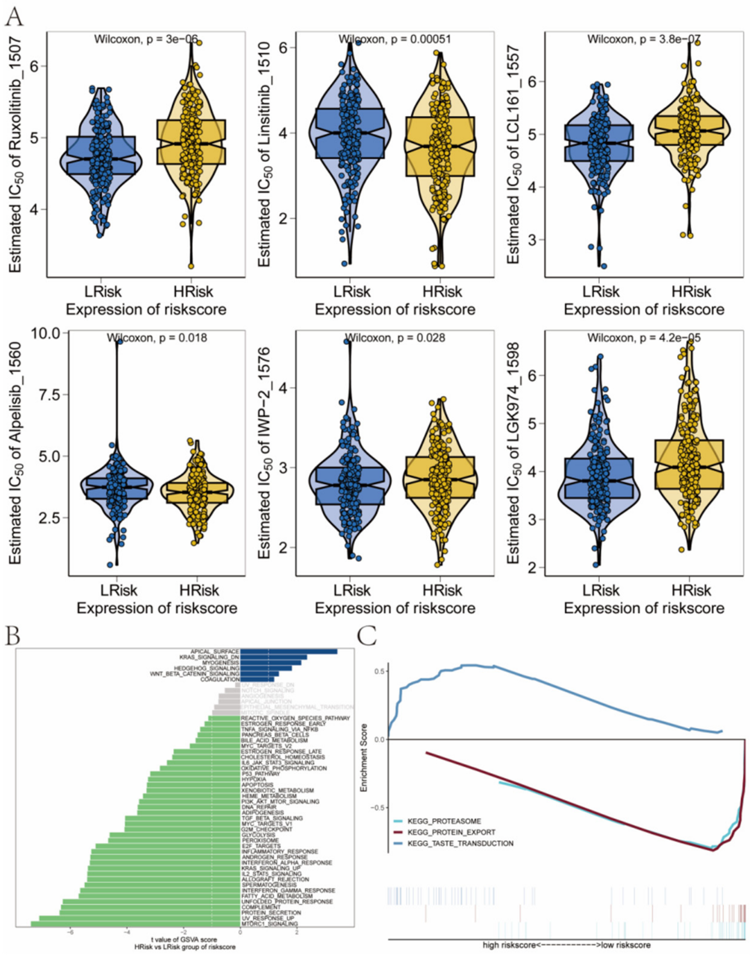

Figure S8. Drug sensitivity and pathway enrichment analyses by risk score. (**A**) Comparison of predicted IC_{50} values for targeted drugs between risk groups. Drug sensitivity was estimated using the oncoPredict R package. Statistical significance was assessed using the Wilcoxon rank-sum test. (**B**) GSVA-based comparison of hallmark pathway activity between risk groups. Enrichment scores were calculated using GSVA and statistical differences were evaluated using the Wilcoxon rank-sum test with FDR correction. (**C**) GSEA of representative KEGG pathways associated with risk scores. Enrichment significance was assessed using permutation testing with FDR correction. All statistical analyses were performed with adjusted P value < 0.05 considered significant.
